# First‐Generation *TTR* Silencing Therapies in Hereditary Transthyretin Amyloidosis With Polyneuropathy: Real‐World Insights From a German Single‐Referral Center

**DOI:** 10.1111/ene.70529

**Published:** 2026-02-18

**Authors:** Marilin S. Koch, Fabian aus dem Siepen, Ute Hegenbart, Stefan Schönland, Markus Weiler

**Affiliations:** ^1^ Amyloidosis Center Heidelberg University Hospital, and Faculty of Medicine, Heidelberg University Heidelberg Germany; ^2^ Department of Neurology Heidelberg University Hospital, and Faculty of Medicine, Heidelberg University Heidelberg Germany; ^3^ Department of Cardiology Heidelberg University Hospital, and Faculty of Medicine, Heidelberg University Heidelberg Germany; ^4^ Department of Medicine V Heidelberg University Hospital, and Faculty of Medicine, Heidelberg University Heidelberg Germany

**Keywords:** ATTRv amyloidosis, Germany, inotersen, patisiran, *TTR* silencer

## Abstract

**Background:**

Hereditary transthyretin (ATTRv) amyloidosis is a rare, genetically heterogeneous disease leading to an unstable configuration of transthyretin (TTR) and hence irregular deposition of TTR amyloid fibrils. The disease predominantly affects the peripheral nervous system, resulting in a progressive sensorimotor polyneuropathy (ATTRv‐PN), but can also involve the cardiac and other organ systems. The *TTR* mRNA silencers patisiran and inotersen effectively reduce serum TTR levels, thereby improving neurologic disability and quality of life in patients with ATTRv‐PN. This real‐world study investigated long‐term efficacy and tolerability of both compounds in patients with ATTRv‐PN.

**Methods:**

This retrospective study from a German single‐referral center assessed the effects of treatment with patisiran or inotersen on clinical parameters of 35 patients with ATTRv‐PN treated at the Amyloidosis Center of Heidelberg University Hospital for up to 6 years after therapy start. The study included analyses of serum TTR levels, NT‐proBNP, creatinine and modified body mass index (mBMI), and reported side effects throughout the observational period.

**Results:**

Both silencers stably reduced serum TTR levels by 81.1% (*p* = 0.0039, patisiran) and 82.8% (*p* = 0.0039, inotersen), respectively, after 8 to 15 months compared to mean baseline. NT‐proBNP, creatinine, and mBMI values remained stable during treatment. With patisiran, a lower rate of reported adverse effects was observed. Grade 1 thrombocytopenia was reported primarily for inotersen‐treated patients.

**Conclusions:**

Our findings confirm long‐term treatment with *TTR* silencers in a real‐world setting is safe, consistently reducing serum TTR levels, stabilizing neurologic symptoms, cardiac and renal functions, and nutritional parameters.

## Graphical abstract

Real‐world long‐term treatment with the first‐generation *TTR* silencers, patisiran and inotersen, in patients with ATTRv‐PN is safe, consistently reducing serum TTR levels, stabilizing neurologic symptoms, cardiac and renal functions, and nutritional parameters.

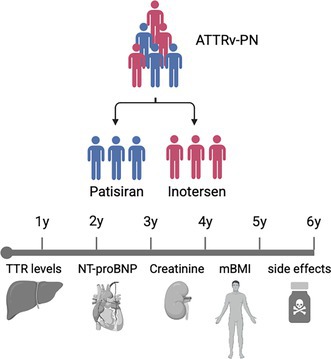

**Abbreviations**
ASO: Antisense oligonucleotide
ATTRv: Amyloid transthyretin variant
ATTRv‐PN: Hereditary transthyretin amyloidosis with polyneuropathy
CTCAE: Common Terminology Criteria for Adverse Events
EMA: European Medicines Agency
FAP: Familial Amyloid Polyneuropathy
FDA: Food and Drug Administration
mBMI: modified Body Mass Index
mNIS+7: modified Neuropathy Impairment Score plus 7 additional assessments
mRNA: messenger ribonucleic acid
NIS: Neuropathy Impairment Score
NT‐proBNP: N‐terminal pro‐B‐type Natriuretic Peptide
NYHA: New York Heart Association
PND: Polyneuropathy disability score
siRNA: small interfering ribonucleic acid
TTR: transthyretin

## 1 Introduction

Hereditary transthyretin (ATTRv) amyloidosis is a rare autosomal‐dominant, primary progressive disease. If untreated, the survival time from symptom onset is 2–15 years [1, 2]. It is caused by variants in the *transthyretin* (*TTR*) gene. *TTR* encodes for a thyroid hormone and retinol‐binding protein/vitamin A carrier protein produced in the liver [3]. TTR variants disrupt the stability of its three‐dimensional protein configuration, culminating in the deposition of extracellular amyloid fibrils, leading to destruction of nearby structures [4, 5]. Besides the heart, the disease primarily affects the peripheral and autonomic nervous system but can also involve, to a lesser extent, the renal and gastrointestinal systems [6]. Depending on the primarily affected organ, ATTRv amyloidosis is clinically classified into a neurologic (presenting with polyneuropathy), a cardiac (presenting with cardiomyopathy), or a mixed phenotype with involvement of both organ systems [7, 8, 9].

Existing therapeutic strategies comprised orthotopic liver transplantation or treatment with the TTR stabilizers tafamidis or diflunisal [10, 11, 12, 13]. Recently, the therapeutic armory for disease modification has been extended to the *TTR* silencing drugs patisiran and inotersen that target *TTR* mRNA, thereby preventing its translation to the protein level.

Patisiran is a small interfering RNA (siRNA) encapsulated in a lipid nanoparticle formulation for targeted hepatic delivery and administered intravenously. It has been investigated for treatment of ATTRv amyloidosis with polyneuropathy (ATTR‐PN) in the phase 3 APOLLO A trial over 18 months [14], showing reduced disease progression, enhanced neurologic status and quality of life while being well tolerated.

An alternative approach to reduce serum TTR levels systemically is the clinically approved treatment with the antisense oligonucleotide (ASO) inotersen. This compound has been evaluated in the phase 3 NEURO‐TTR trial, demonstrating slower disease progression and improved quality of life but requiring regular laboratory monitoring for signs of glomerulonephritis and thrombocytopenia [15].

As the follow‐up period for all *TTR* silencers in the clinical trial setting was limited to 15–18 months, these datasets do not provide evidence on the effects of long‐time treatment. Therefore, the aim of this retrospective study was to overcome this information gap by examining long‐term efficacy and tolerability of both patisiran and inotersen in a real‐world setting. The primary endpoint of this study was the change from baseline serum TTR levels after 8–15 months of therapy. Secondary endpoints were baseline changes for N‐terminal pro‐B‐type natriuretic peptide (NT‐proBNP), serum creatinine levels, modified body mass index (mBMI), and the neuropathy impairment score (NIS) within the same time frame.

## 2 Methods

### 2.1 Study Design

This study was designed as a retrospective observational single‐referral center analysis for the treatment effects and safety of patisiran and inotersen in patients with genetically proven ATTRv‐PN, conducted at the Amyloidosis Center of Heidelberg University Hospital. Approval of this study was granted by the ethics committee of the Medical Faculty of Heidelberg University (123/2006). Inclusion criteria were a clinically manifest ATTRv‐PN and treatment with either patisiran or inotersen. Patients who received both treatments sequentially were attributed to the cohort of their first‐line therapy and censored for functional parameter analysis, i.e., TTR, NT‐proBNP, serum creatinine, mBMI, and NIS, after switch of therapy. Due to the retrospective nature, data were collected at predefined time frames depending on the assessed clinical parameters. Enrollment started in close temporal proximity to the initiation of therapy with either patisiran or inotersen with the earliest silencer treatment beginning in January 2018. Database lock‐date was March 31, 2024. The last patient visit was in February 2024. Cut‐off for median follow‐up of all patients regardless of therapy switch was March 31, 2024. Patients were censored after beginning of second generation silencer treatment with vutrisiran. Baseline parameters were defined as those obtained directly before start of therapy; if no value was recorded at that time point, the most recent prior measurement was defined as baseline if not obtained 5 months or more before therapy start.

Pharmacological efficacy was ascertained with serum TTR/prealbumin levels. A possible influence of the treatment on cardiac and renal involvement was evaluated with the respective laboratory parameters NT‐proBNP and creatinine. Treatment impact on the nutritional state was determined using the mBMI which is commonly used as an indicator for nutritional status in patients with amyloidosis as it corrects for potential inaccurate weight measurements due to fluid retention and acts as a prognostic factor in ATTRv amyloidosis [16, 17]. If not indicated otherwise, all laboratory parameters are given as mean ± SD.

Neurologic impairment at treatment start was assessed applying the FAP stage [18] and the Peripheral Neuropathy Disability (PND) score [19]. If no FAP stage or PND score was documented, it was determined retrospectively based on the available medical history and neurologic examination. Neurologic progression throughout the data collection period was analyzed utilizing the NIS [20], if available; patients were included if at least ≥ 3 values were recorded at baseline and follow‐up period. As the NIS had been documented inconsistently, it could not be provided for every patient and/or timepoint.

### 2.2 Statistical Analysis

Statistical analysis was taken out with GraphPad Prism (version 10). Testing for normality was performed with the Shapiro–Wilk test, showing predominantly non‐normal data distribution. Therefore, comparison of baseline with follow‐up values was performed with the Wilcoxon matched‐pairs signed rank test using Bonferroni correction. Comparison of serum creatinine levels at baseline between patisiran and inotersen patient cohorts was performed with the Mann–Whitney test. In a second step, adjustment for relevant confounders was executed with a multiple linear regression analysis.

## 3 Results

### 3.1 Study Population

**FIGURE 1:**
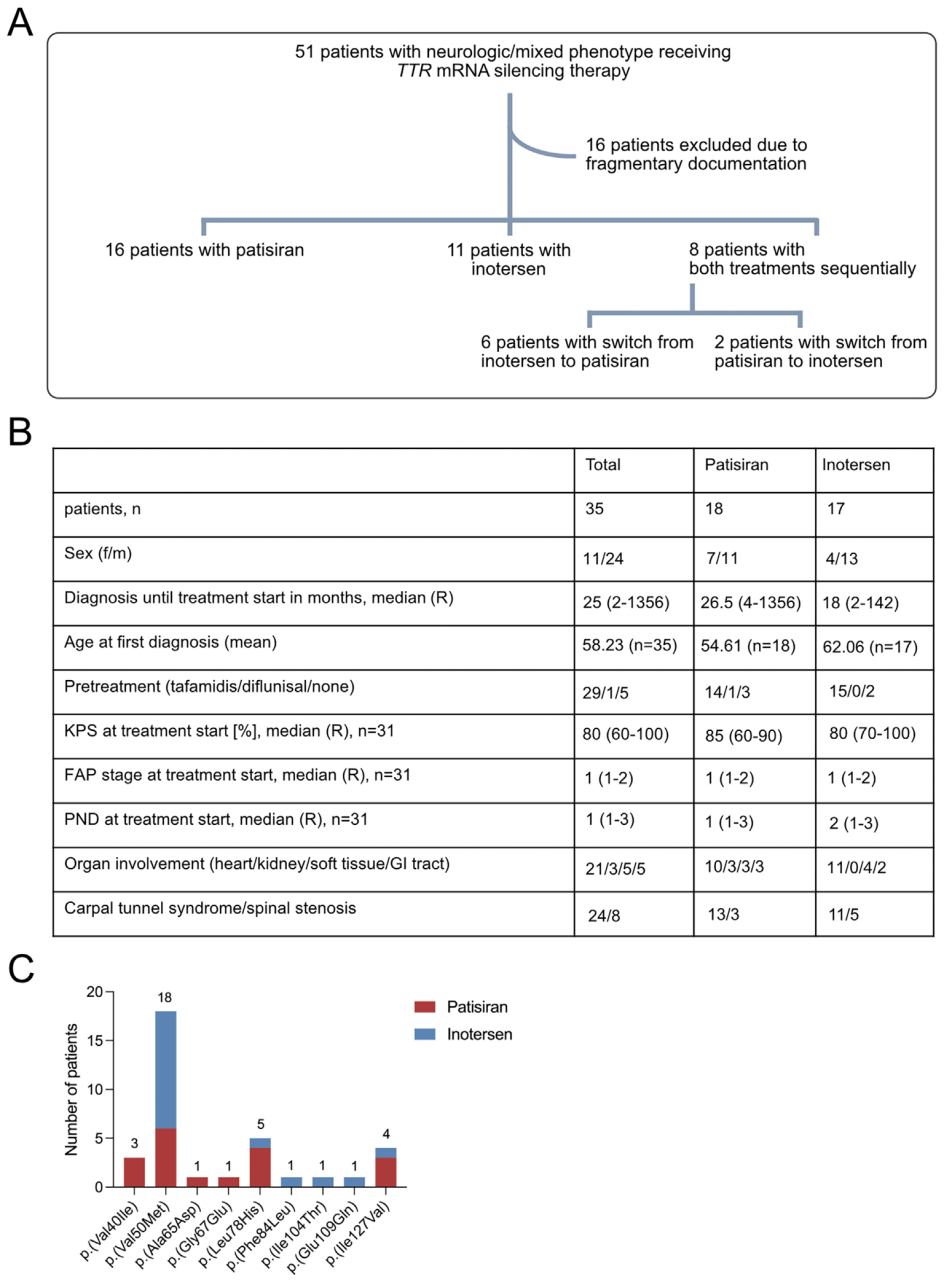
(A) Overview of the study population. (B) Patient characteristics of the total, patisiran, and inotersen cohorts at baseline. (C) Overview of all TTR variants across the study population and the distribution of applied *TTR* silencers. Numbers above bars indicate count of patients per TTR variant.

A total of 51 patients with neurologic or mixed phenotype receiving *TTR* silencing treatment with either patisiran or inotersen were identified within the ATTRv amyloidosis patient cohort at the Amyloidosis Center of Heidelberg University Hospital. Of those, 16 patients were excluded from the analysis due to fragmentary or incomplete documentation. Among the remaining patients, 16 patients received patisiran, 11 patients received inotersen, and 8 patients were treated with both patisiran and inotersen sequentially (Figure 1A). After switch of therapy, patients were assigned to the cohort of their first‐line therapy.

### 3.2 Patient Characteristics

Patient characteristics are given in detail in Figure 1B. Twenty‐four patients receiving *TTR* silencing treatment were male (*n* = 24/35, 68.6%), while the proportion of female patients was lower (*n* = 11/35, 31.4%). The majority of female patients was treated with patisiran (*n* = 7/11, 63.6%) in contrast to inotersen (*n* = 4/11, 36.4%). Among male patients, treatments were approximately evenly distributed (patisiran: *n* = 11/24, 45.83%; inotersen: *n* = 13/24, 54.2%).

The mean age at first diagnosis for the whole patient cohort was 58.23 ± 11.29 (mean ± STD) years. Patients receiving patisiran (54.61 ± 10.68 years) were younger than patients receiving inotersen (62.06 ± 10.93 years). The median time from first diagnosis to start of *TTR* silencing therapy was 26.5 months for patisiran and 18 months for inotersen. Patients presented with a moderate overall health status at the beginning of therapy in both treatment groups, as determined by a Karnofsky Performance score ≥ 80%.

The majority of patients had received pre‐treatment with tafamidis (*n* = 29/35, 82.9%); one patient had been treated with diflunisal (*n* = 1/35, 2.9%), and a small proportion of patients were therapy‐naïve (*n* = 5/35, 14.3%). This was reflected accordingly in the patisiran (tafamidis: *n* = 14/18, 77.8%, diflunisal: *n* = 1/18, 5.6%, no therapy: *n* = 3/18, 16.7%) and inotersen (tafamidis: *n* = 15/17, 88.2%, no therapy: *n* = 2/17, 11.8%) treatment groups.

Neurologically, the severity of polyneuropathy within the patient cohort was limited (FAP stage = 1 and PND = I‐II for all treatment groups), reflecting the approval criteria for both patisiran and inotersen in Germany. The majority of patients additionally presented with carpal tunnel syndrome (*n* = 24) and were treated more often with patisiran (*n* = 13/24, 54.2%) than with inotersen (*n* = 11/24, 45.8%). Occurrence of spinal stenosis was documented for fewer patients (*n* = 8); here more patients were treated with inotersen (*n* = 5/8, 62.5%) than with patisiran (*n* = 3/8, 37.5%). While FAP stage and PND score are based on walking capacity, the NIS additionally includes a detailed functional sensorimotor assessment. In the patisiran treatment group, the NIS for the single patients remained fairly stable over time, except for one (asterisk). The inotersen group presented with similar results, with two patients showing an increased NIS (asterisk). As NIS data availability across patients (data available only for 8 patients treated with patisiran and 6 patients treated with inotersen) and timepoints (minimum 3 to maximum 7 timepoints) was limited, the analysis had to remain descriptive (Figure S1A).

Apart from neurologic involvement, a predominant share of patients presented with cardiac involvement (*n* = 21/35, 60%), while the impact on the renal system (*n* = 3/35, 85.7%), soft tissue (*n* = 5/35, 14.3%), and gastrointestinal system (*n* = 5/35, 14.3%) was considerably lower. Within the patisiran and inotersen treatment cohorts, organ involvement showed a similar distribution pattern, with the exception that no patient with renal amyloidosis received inotersen.

Within the ATTRv patient cohort receiving *TTR* silencing treatment, p.(Val50Met) (*n* = 18/35, n 51.4%) was the predominant variant, followed by p.(Leu78His) (*n* = 5/35, 14.3%), p.(Ile127Val) (*n* = 4/35, 11.4%), and p.(Val40Ile) (*n* = 3/35, 8.6%) (Figure 1C). While patients with the most common variant p.(Val50Met) more frequently received inotersen treatment (*n* = 12/18, 66.7%), patisiran was mainly administered to patients harboring p.(Leu78His) (*n* = 4/5, 80%), p.(Ile127Val) (*n* = 3/4, 75%), or p.(Val40Ile) (*n* = 3/3, 100%) (Figure 1C).

During the observational period, 28 patients switched to the second‐generation silencer vutrisiran (*n* = 28/35, 80%); seven patients (*n* = 7/35, 20%) adhered to their original treatment (*n* = 5 patisiran, *n* = 2 inotersen). Switching to vutrisiran occurred between 11/2022 and 01/2024, and patients were censored from analyses after the switch to vutrisiran.

### 3.3 Both Patisiran and Inotersen Treatments Are Associated With Consistent Serum TTR Reduction

**FIGURE 2:**
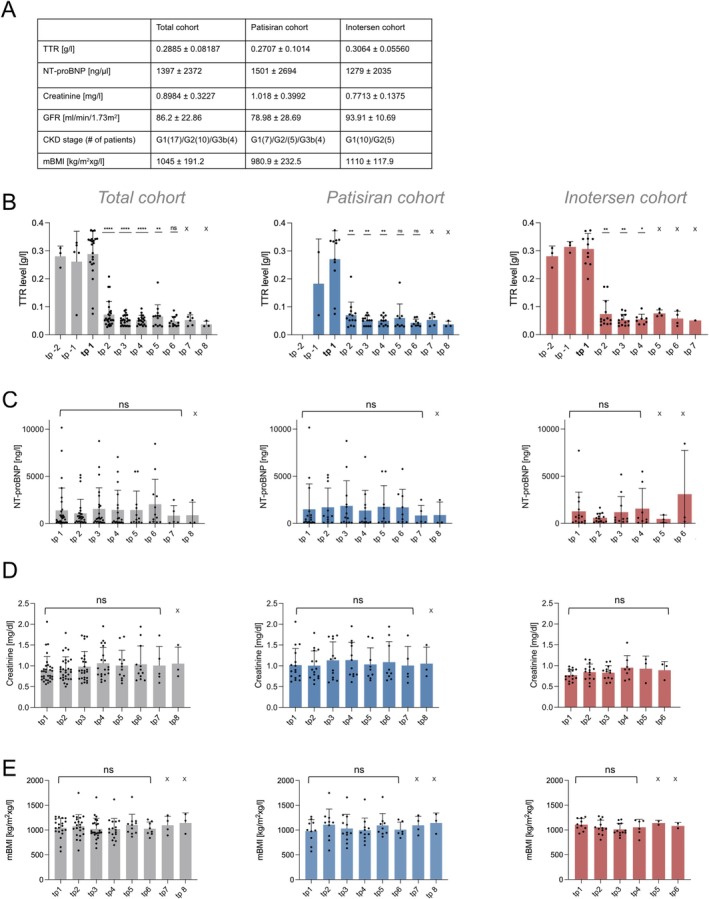
(A) Laboratory baseline characteristics by treatment group. Values are displayed as mean ± standard deviation. (B) Compared to baseline, significantly reduced serum TTR levels for up to 3 years in the total *TTR* silencer cohort were observed (Wilcoxon matched‐pair signed rank test: Baseline vs. 3–7 months: *P* < 0.0001, vs. 8–15 months *p* < 0.0001, vs. 2 years *p* = 0.0001, vs. 3 years *p* = 0.0078, vs. 4 years *p* = 0.0156; due to small sample size no statistical testing could be performed for 5 and 6 years. Bonferroni corrected threshold: *P* < 0.01) and for up to 2 years for patisiran (Wilcoxon matched‐pair signed rank test: Baseline vs. 3–7 months: *P* = 0.0039, vs. 8–15 months *p* = 0.0039, vs. 2 years *p* = 0.0078, vs. 3 years *p* = 0.0312, vs. 4 years *p* = 0.0625; due to small sample size no statistical testing could be performed for 5 and 6 years (marked by X). Bonferroni corrected threshold: *P* < 0.01) and 8–15 months for the inotersen cohort (Wilcoxon matched‐pairs signed rank test: Baseline vs. 3–7 months: *P* = 0.0039, vs. 8–15 months *p* = 0.0039, vs. 2 years p = 0.0312, due to small sample size no statistical testing could be performed for 3, 4, and 5 years. Bonferroni corrected threshold: *P* < 0.0166). TTR levels continued to be lower than baseline after 2 years after start of therapy, although statistical significance was not reached, possibly due to small sample size. TTR levels were gathered at the following timepoints: Tp −2 and −1: 2 and 1 year before start of therapy, tp 1: Baseline, tp 2: 3–7 months, tp 3: 8–15 months, tp 4: 2 years, tp 5: 3 years, tp 6: 4 years, tp 7: 5 years after start of therapy. Number of patients included per cohort: Total *n* = 34, patisiran *n* = 18, inotersen *n* = 16. (C‐E) Compared to mean baseline, NT‐proBNP (*p*‐values all: 0.1828–0.9781, *p*‐values patisiran 0.1523–0.8750, *p*‐values inotersen: 0.6377–0.8438), creatinine (*p*‐values all: 0.1707‐ > 0.999, *p*‐values patisiran 0.2821–0.9844, *p*‐values inotersen: 0.1094–0.5), and mBMI levels (*p*‐values all: 0.0625–0.3757, *p*‐values patisiran 0.125–0.9453, *p*‐values inotersen: 0.0781–0.5469) remained stable throughout the course of therapy. At later timepoints (marked by X), statistics were not possible due to small sample size. NT‐proBNP, creatinine, and mBMI levels were collected at the following timepoints: Tp 1: Baseline, tp 2: 3–8 months, tp 3: 9–15 months, tp 4: 2 years, tp 5: 3 years, tp 6: 4 years, tp 7: 5 years, tp 8: 6 years after start of therapy. Number of patients included per cohort: Total (NT‐proBNP/creatinine/mBMI) *n* = 35/35/34, patisiran (NT‐proBNP/creatinine/mBMI) *n* = 18/18/18, and inotersen (NT‐proBNP/creatinine/mBMI) *n* = 17/17/16.

To confirm activity of the respective treatment, serum TTR levels before start of therapy (if available) and after were analyzed for long‐term suppression of TTR. We observed a consistent and significant serum TTR reduction for the total *TTR* silencer cohort for up to 3 years (adj. *p* < 0.01 for baseline vs. 3–7 months, 8–15 months, 2, and 3 years after start of therapy) and for the single patisiran (adj. *p* < 0.01 for baseline vs. 3–7 months, 8–15 months, and 2 years after start of therapy) and inotersen cohorts (adj. *p* < 0.0166 for baseline vs. 3–7 months, and 8–15 months) for up to 2 years resp. 8–15 months after start of therapy. While TTR levels were lower than baseline after 2 years of therapy with both patisiran and inotersen and after 3 years within the total cohort, statistical significance was not met, likely due to small sample size (Figure 2B). Serum TTR levels were reduced by 81.1% of the mean baseline value at therapy start in response to treatment with patisiran (0.2707 ± 0.1014 g/L vs. 0.05117 ± 0.01647 g/L) and by 82.8% in response to treatment with inotersen (0.3064 ± 0.05560 g/L vs. 0.05266 ± 0.02012 g/L) after 8–15 months of therapy. A similar steady state of serum TTR levels was then observed for the following timepoints for both compounds (patisiran ranging from 0.06031 ± 0.05009 to 0.03760 ± 0.01076 g/L, inotersen ranging from 0.07625 ± 0.01234 to 0.05100 ± 0 g/L) (Figure 2A).

### 3.4 Stable Cardiac and Renal Functions and Parameters in Response to *TTR* Silencing Treatment

To dissect a possible effect of *TTR* silencing treatments on cardiac and renal parameters, we assessed serum creatinine and NT‐proBNP levels throughout the course of therapy.

Within the total patient cohort, mean NT‐proBNP levels ranged from 835 ± 1070 ng/L to 2046 ± 2647 ng/L. NT‐proBNP levels ranged from 835 ± 1070 ng/L to 1860 ± 2662 ng/L in the patisiran, and from 472.3 ± 403.6 ng/L to 3090 ± 4652 ng/L in the inotersen group (Figure 2C).

Mean creatinine levels ranged from 0.8984 ± 0.3227 mg/dL to 1.063 ± 0.3807 mg/dL for all patients. In principle, we observed higher mean creatinine levels in patients treated with patisiran (ranging from 1.005 ± 0.3527 mg/dL to 1.138 mg/dL ±0.4261 mg/dL) than with inotersen (ranging from 0.7713 ± 0.1375 mg/dL to 0.9500 ± 0.2897 mg/dL) at baseline (patisiran: 1.018 ± 0.3992 mg/dL vs. inotersen: 0.7713 ± 0.1375 mg/dL), although this difference was not statistically significant in unadjusted testing (Mann–Whitney test, *p* = 0.0733) (Figure 2D, Figure S1B). However, after adjustment for age, sex, genotype, cardiac involvement, and time from diagnosis to therapy, *TTR* silencer treatment was a significant predictor of creatinine levels (multiple linear regression analysis, *p* = 0.0029), and also cardiac involvement showed an independent association with creatinine levels (multiple linear regression analysis, *p* = 0.0286) (Figure S1C). These results suggest that confounders may have masked the effect in the unadjusted comparison. Compared with the serum levels at start of therapy, both NT‐proBNP and creatinine levels remained stable as no statistically significant change of these parameters was observed.

### 3.5 Treatment With Patisiran or Inotersen Is Associated With a Stable Nutritional State

mBMI values during the course of therapy were analyzed for a potential therapeutic impact. At baseline, the mean mBMI was above the normal range with > 900 kg/m^2^xg/l for the overall patient cohort (1045 ± 191.2 kg/m^2^xg/l), and the patisiran (980.9 ± 232.5 kg/m^2^xg/l) and inotersen treatment cohorts (1110 ± 117.9 kg/m^2^xg/l). Comparison of baseline mBMI to follow‐up values for all treatment conditions showed no significant change, indicating a stable nutritional situation (Figure 2E).

### 3.6 Less Adverse Side Effects on Patisiran Treatment

**FIGURE 3:**
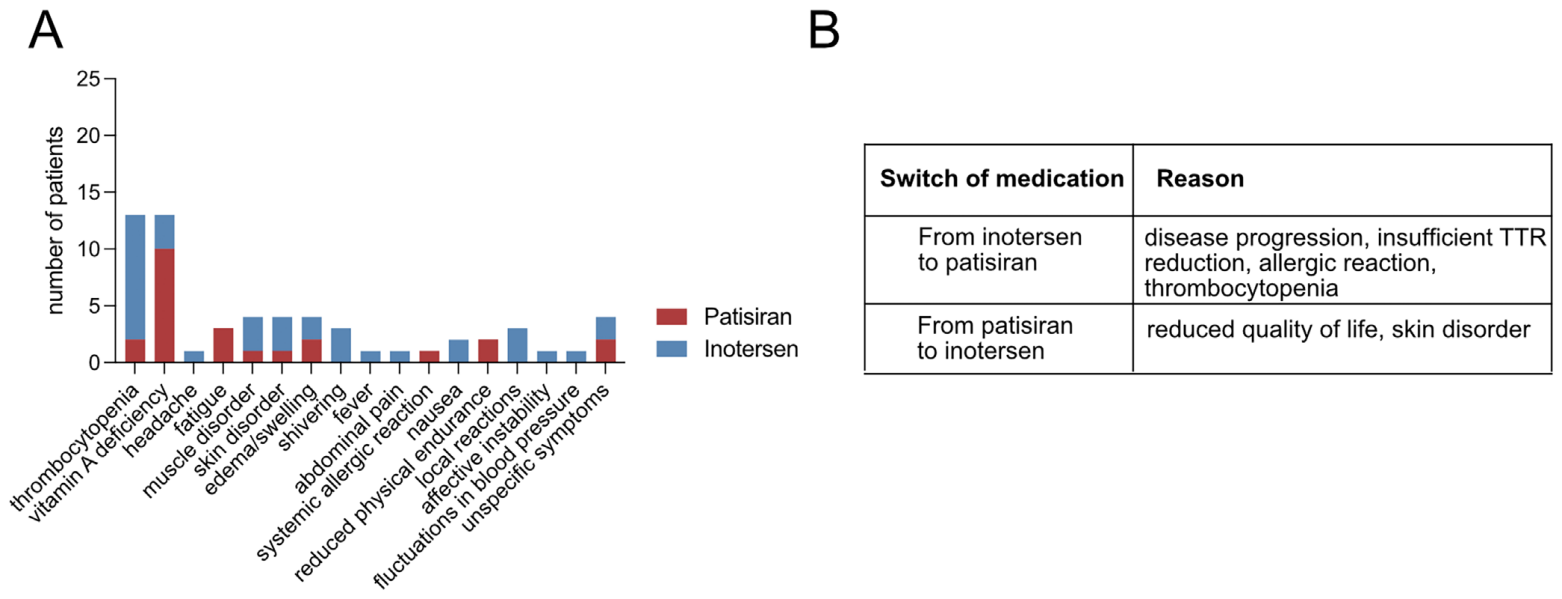
(A) Overview of documented therapy‐related adverse side effects. (B) Reasons for switch of medication.

In comparison, patisiran treatment was associated with less adverse side effects than inotersen. Only seven patients (*n* = 7/35, 20%) did not present with adverse effects (patisiran *n* = 5, inotersen *n* = 2). The most common documented therapeutic complications were vitamin A deficiency and thrombocytopenia. 13 patients presented with thrombocytopenia (37.1%), the majority of these patients received inotersen (*n* = 11/13, 84.6%), while two patients (*n* = 2/13, 15.4%) were treated with patisiran. For three patients, thrombocytopenia occurred before und during the switch from inotersen to patisiran; therefore, thrombocytopenia was attributed to inotersen. For all patients, thrombocytopenia did not exceed CTCAE grade 1. In contrast, vitamin A deficiency was observed in 12 patients; mainly in the cohort treated with patisiran (*n* = 10/12, 83.3%) and less frequently in inotersen‐treated patients (*n* = 2/12, 16.7%). One patient presented with reduced vitamin A levels on both patisiran and inotersen treatment. Other adverse effects included muscular disorders including myalgia, weakness or cramps (*n* = 1 patisiran, *n* = 3 inotersen), edema/swelling (*n* = 2 patisiran, *n* = 2 inotersen) and unspecific symptoms, e.g., discomfort (*n* = 3 patisiran, *n* = 1 inotersen) as well as skin disorders (*n* = 1 patisiran, *n* = 3 inotersen) such as exanthema/rash and vitamin A deficiency‐related skin changes. One patient presented with signs of a systemic allergic reaction, i.e., shortness of breath and pruritus. A detailed summary of the documented side effects and their distribution among the treatment groups is displayed in Figure 3A.

Decline of quality of life and skin disorder were the main reasons for a treatment switch from patisiran to inotersen, while the rationales for a transition from inotersen to patisiran were disease progression, insufficient serum TTR reduction, allergic reaction, or thrombocytopenia (Figure 3B).

### 3.7 Overall Survival

Median follow‐up time from start of therapy with *TTR* silencers until database lock date was 58 months for all patients (including those who had switched to vutrisiran) and 53 months excluding those patients who had switched to vutrisiran in the meantime. Until then, 34 patients were alive (97%), and one patient had died of amyloidosis‐related complications.

## 4 Discussion

The siRNA drug patisiran and the ASO drug inotersen both disrupt hepatic TTR production by interfering with *TTR* mRNA. Both compounds have been shown to effectively reduce serum TTR levels, thus stabilizing neurologic disability and enhancing quality of life in patients with ATTRv‐PN [14, 15]. With this monocentric retrospective real‐world analysis, we provide evidence that treatment with either of the *TTR* silencers, patisiran or inotersen, is well tolerated beyond the monitoring period of the pivotal clinical trials. We demonstrate a consistent reduction of serum TTR levels and stable disease‐defining functional parameters.

The APOLLO phase three clinical trial investigated the efficacy and safety of patisiran compared to placebo in patients with ATTRv‐PN stage 1 or 2 for a total of 18 months with infusions every three weeks. The primary endpoint was neurologic disability, as measured with the mNIS+7. 56% of patients who received patisiran presented with improved neuropathy. While 54% showed a moderate increase in mNIS+7, this was considerably lower than in patients who received placebo. Quality of life was enhanced in 51% of patisiran‐treated patients, compared to 10% in the placebo group [14]. Inotersen was evaluated in the NEURO‐TTR phase 3 clinical trial. After three initial injections, patients received inotersen or placebo subcutaneously on a weekly basis. Among the inotersen‐treated patients, progression of neuropathy and degradation of quality of life were significantly reduced compared to placebo [15]. These results clearly demonstrated that both *TTR* silencers, patisiran or inotersen, positively influence the natural course of ATTRv‐PN. However, these results were obtained from homogenous patient cohorts analyzed in controlled clinical trials conducted in a limited time‐frame. In this context, real‐world data can complement those studies by supplying information on long‐term efficacy and safety in heterogenous patient collectives that do not follow a predefined trial protocol.

In the present retrospective study, treatment with either patisiran or inotersen resulted in consistent and durable reduction of serum TTR levels at 8–15 months, and the primary endpoint of the study was thus met. No statistically significant change of NT‐proBNP, serum creatinine, or mBMI values was noted throughout the course of therapy with either patisiran or inotersen, indicating a stabilizing treatment effect on cardiac and renal functions and the nutritional status throughout the retrospective monitoring period of up to six years. The disease‐stabilizing effects of *TTR* silencer treatment in our study are consistent with findings from other groups. In the “patisiranItaly”, a multicenter retrospective study (cohort size 181 patients, follow‐up period 36 months) [21], stable NT‐proBNP levels and stable neurologic status in 43.6% of patients were observed, and treatment was well‐tolerated. Similar results for patisiran in a real‐world setting were obtained in a study of a Belgian patient collective (cohort size 31 patients, 12 patients treated with patisiran, and follow‐up period 31 months) [22], reporting predominantly unaltered FAP and NYHA stages—and by another Italian study, showing disease stability including the neurologic and nutritional status and quality of life (cohort size 40 patients; follow‐up period 48 months) [23]. A stabilizing effect on renal function was also previously reported for inotersen in the NeuroTTR follow‐up study that monitored ten patients of the original trial for up to 18–76 months after treatment start [24]. In our study, we observed higher, but not statistically significant mean serum creatinine levels in the patisiran cohort compared to inotersen at baseline. After confounder adjustment, *TTR* silencing treatment with either patisiran or inotersen was shown to be a significant predictor of serum creatinine levels. This association might be explained by the fact that inotersen may produce adverse renal side effects; therefore, patients with preexisting renal dysfunction had received patisiran. Serum creatinine may underestimate renal impairment in ATTRv amyloidosis because sarcopenia and low muscle mass can lower creatinine levels independent from kidney function. Therefore, future studies should incorporate cystatin C or measured GFR.

The adverse event profile of both compounds was within a tolerable range. Whereas patisiran treatment in the pivotal trial was predominantly associated with infusion‐related side effects [14], adverse events across inotersen‐treated patients included glomerulonephritis and thrombocytopenia [15]. Correspondingly, the rate of adverse events in this retrospective observational study was higher in the inotersen cohort. While the median time from first diagnosis to the initiation of a *TTR* silencing therapy was slightly longer in the patisiran cohort (26.5 vs. 18 months), patients in the inotersen group were older, making an age‐related bias possible in this context.

The main limitation of our study is its retrospective approach. In contrast to prospective studies, the medical information provided in this study does not claim to be complete. This particularly affects the rating of neurologic symptoms with the NIS, which was not regularly assessed in routine clinical practice within the patient cohort at hand; therefore, NIS was only available in a restricted number of patients and time points, and its analysis had to remain descriptive. Furthermore, our study has a preponderance of male participants. However, this is indicative of the epidemiology of the disease affecting men, especially with cardiac involvement, more frequently than women [25].

The primary strength of our study is its extensive observational period of up to 6 years, enabling assessments on long‐term efficacy and tolerability of *TTR* silencing medications. Moreover, our data provide a basis for a comparison of the two *TTR* silencing strategies, siRNA and ASO, in a real‐world patient collective with a wide variety of *TTR* variants, reflecting the genetic heterogeneity of ATTRv amyloidosis.

Recently, the second‐generation siRNA drug vutrisiran [26] and the ASO drug eplontersen [27] were approved for treatment of ATTRv‐PN, demonstrating improved neurologic status and quality of life, while having an adequate safety profile. Whereas both patisiran and inotersen have to be administered every three weeks intravenously or once a week subcutaneously, respectively, vutrisiran and eplontersen are injected subcutaneously every three months or once a month, respectively, thus reducing the therapeutic burden and time commitment for patients.

Collectively, this study indicates beyond the pivotal trials of both patisiran and inotersen that *TTR* silencers are well tolerated and effectively reduce serum TTR levels in patients with a neurologic or mixed phenotype while stabilizing relevant disease‐defining functional parameters in a long‐term real‐world setting.

## Author Contributions

**Marilin S. Koch:** writing – original draft, visualization, writing – review and editing, investigation, formal analysis, project administration, data curation, conceptualization. **Fabian aus dem Siepen:** writing – review and editing. **Ute Hegenbart:** conceptualization, data curation, writing – review and editing, supervision. **Stefan Schönland:** conceptualization, writing – review and editing, supervision. **Markus Weiler:** conceptualization, writing – review and editing, project administration, investigation, formal analysis, supervision.

## Funding

The authors have nothing to report.

## Conflicts of Interest

Marilin S. Koch reports no disclosures relevant to this work. Fabian aus dem Siepen reports advisory board and speaker honoraria from Akcea Therapeutics, Alnylam Pharmaceuticals, Bayer, and Pfizer outside this work. Stefan Schönland reports travel grant, honoraria, and research funding from Janssen, Neurimmune and Prothena; received research funding from Sanofi; received honoraria from Pfizer and Takeda; and is an adviser for Telix; received travel grants from Binding Site, Celgene, and Jazz. Ute Hegenbart reports travel grants from Janssen, Prothena, and Pfizer, advisory board honoraria from Pfizer, Prothena, and Neuroimmune, honoraria from Janssen, Pfizer, Alnylam Pharmaceuticals, and Akcea Therapeutics, and financial support for the Amyloidosis Registry from Prothena and Janssen outside this work. Markus Weiler reports advisory board or consultant honoraria from Akcea Therapeutics, Alnylam Pharmaceuticals, Biogen, F. Hoffmann‐La Roche, Novartis, Novo Nordisk, Pfizer, Purpose Pharma, and Swedish Orphan Biovitrum AB; speaker fees from Akcea Therapeutics, Alnylam Pharmaceuticals, AstraZeneca, and Biogen; and financial support for conference or meeting attendances from Akcea Therapeutics, Alnylam Pharmaceuticals, AstraZeneca, BridgeBio, Ionis, and Pfizer; outside this work.

## Supporting information


**Figure S1:** (A) NIS during the course of therapy. Each line presents the temporal development of the NIS per patient. Data were obtained at the following timepoints: tp −2: 2–2.5 years pretreatment, tp −1: 1–1.5 years pre‐treatment, tp 1: baseline, tp 2: 3–7 months, tp 3: 9–15 months, tp 4: 16–21 months, tp 5: 22–27 months, tp 6: 28–31 months, tp 7: 33–38 months, tp 8: 40–42 months, tp 9: 44–48 months, tp 10: 51–53 months, tp 11: 54–57 months, tp 12: 59–60 months, tp 13: 65 months, tp16: 71 months after start of therapy.|
(B) Patients treated with patisiran showed higher serum creatinine levels at baseline than patients treated with inotersen, but this difference was not statistically significant (Mann–Whitney‐test, *p* = 0.0733), patisiran *n* = 16, inotersen *n* = 15.
(C) Multiple linear regression analysis for serum creatinine levels. Analysis demonstrates that therapy, i.e., patisiran or inotersen, was a significant predictor of creatinine levels (*p* = 0.0029) after adjustment for sex, genotype, age, cardiac involvement, and time from diagnosis until start of therapy. Additionally, cardiac involvement showed an independent association with serum creatinine levels (*p* = 0.0286).

## Data Availability

The data that support the findings of this study are available from the corresponding author upon reasonable request.

## References

[1] T. Damy , I. Conceição , P. García‐Pavía , et al., “A Simple Core Dataset and Disease Severity Score for Hereditary Transthyretin (ATTRv) Amyloidosis,” Amyloid 28, no. 3 (2021): 189–198, 10.1080/13506129.2021.1931099. 34042016

[2] H. Koike , F. Tanaka , R. Hashimoto , et al., “Natural History of Transthyretin Val30Met Familial Amyloid Polyneuropathy: Analysis of Late‐Onset Cases From Non‐Endemic Areas,” Journal of Neurology, Neurosurgery, and Psychiatry 83, no. 2 (2012): 152–158, 10.1136/JNNP-2011-301299. 22228785

[3] M. A. Liz , T. Coelho , V. Bellotti , M. I. Fernandez‐Arias , P. Mallaina , and L. Obici , “A Narrative Review of the Role of Transthyretin in Health and Disease,” Neurology and Therapy 9, no. 2 (2020): 395–402, 10.1007/S40120-020-00217-0. 33001386 PMC7606379

[4] D. Adams , H. Koike , M. Slama , and T. Coelho , “Hereditary Transthyretin Amyloidosis: A Model of Medical Progress for a Fatal Disease,” Nature Reviews Neurology 15, no. 7 (2019): 387–404, 10.1038/s41582-019-0210-4. 31209302

[5] F. Manganelli , G. M. Fabrizi , M. Luigetti , P. Mandich , A. Mazzeo , and D. Pareyson , “Hereditary Transthyretin Amyloidosis Overview,” Neurological Sciences 43, no. Suppl 2 (2020): 595, 10.1007/S10072-020-04889-2. 33188616 PMC9780126

[6] M. S. Maurer , M. Hanna , M. Grogan , et al., “Genotype and Phenotype of Transthyretin Cardiac Amyloidosis: THAOS (Transthyretin Amyloid Outcome Survey),” Journal of the American College of Cardiology 68, no. 2 (2016): 161–172, 10.1016/J.JACC.2016.03.596. 27386769 PMC4940135

[7] L. Poli , B. Labella , S. Cotti Piccinelli , et al., “Hereditary Transthyretin Amyloidosis: A Comprehensive Review With a Focus on Peripheral Neuropathy,” Frontiers in Neurology 14 (2023): 1242815, 10.3389/FNEUR.2023.1242815. 37869146 PMC10585157

[8] Y. Ando , D. Adams , M. D. Benson , et al., “Guidelines and New Directions in the Therapy and Monitoring of ATTRv Amyloidosis,” Amyloid 29, no. 3 (2022): 143–155, 10.1080/13506129.2022.2052838. 35652823

[9] F. Triposkiadis , A. Briasoulis , R. C. Starling , et al., “Hereditary Transthyretin Amyloidosis (ATTRv),” Current Problems in Cardiology 50, no. 4 (2025): 103019, 10.1016/J.CPCARDIOL.2025.103019. 39954876

[10] D. Adams , D. Samuel , and C. Goulon‐Goeau , “The Course and Prognostic Factors of Familial Amyloid Polyneuropathy After Liver Transplantation,” Brain 123, no. 7 (2000): 1495–1504, 10.1093/BRAIN/123.7.1495. 10869060

[11] T. Coelho , L. F. Maia , and A. M. Da Silva , “Tafamidis for Transthyretin Familial Amyloid Polyneuropathy: A Randomized, Controlled Trial,” Neurology 79, no. 8 (2012): 785–792, 10.1212/WNL.0B013E3182661EB1. 22843282 PMC4098875

[12] Y. Sekijima , M. A. Dendle , and J. W. Kelly , “Orally Administered Diflunisal Stabilizes Transthyretin Against Dissociation Required for Amyloidogenesis,” Amyloid 13, no. 4 (2006): 236–249, 10.1080/13506120600960882. 17107884

[13] P. Socie , A. Benmalek , C. Cauquil , et al., “Comparison Between Tafamidis and Liver Transplantation as First‐Line Therapy for Hereditary Transthyretin Amyloidosis,” Amyloid 30, no. 3 (2023): 303–312, 10.1080/13506129.2023.2177986. 36795029

[14] D. Adams , A. Gonzalez‐Duarte , and W. D. O'Riordan , “Patisiran, an RNAi Therapeutic, for Hereditary Transthyretin Amyloidosis,” New England Journal of Medicine 379, no. 1 (2018): 11–21, 10.1056/NEJMOA1716153. 29972753

[15] M. D. Benson , M. Waddington‐Cruz , J. L. Berk , et al., “Inotersen Treatment for Patients With Hereditary Transthyretin Amyloidosis,” New England Journal of Medicine 379, no. 1 (2018): 22–31, 10.1056/NEJMOA1716793. 29972757 PMC12611561

[16] O. Suhr , A. Danielsson , G. Holmgren , and L. Steen , “Malnutrition and Gastrointestinal Dysfunction as Prognostic Factors for Survival in Familial Amyloidotic Polyneuropathy,” Journal of Internal Medicine 235, no. 5 (1994): 479–485, 10.1111/J.1365-2796.1994.TB01106.X. 8182405

[17] E. Driggin , S. Helmke , J. De Los Santos , et al., “Markers of Nutritional Status and Inflammation in Transthyretin Cardiac Amyloidosis: Association With Outcomes and the Clinical Phenotype,” Amyloid 27, no. 2 (2020): 73–80, 10.1080/13506129.2019.1698417. 31825676

[18] P. Coutinho , A. Martins da Silva , L. J. Lopes , and A. Resende Barbosa , “Forty Years of Experience With Type I Amyloid Neuropathy. Review of 483 Cases,” In Amyloid and Amyloidosis, ed. G. G. Glenner , P. Pinho a Costa , and F. de Freitas , (Excerpta Medica, 1980), 88–98.

[19] S. Yamamoto , H. E. Wilczek , G. Nowak , et al., “Liver Transplantation for Familial Amyloidotic Polyneuropathy (FAP): A Single‐Center Experience Over 16 Years,” American Journal of Transplantation 7, no. 11 (2007): 2597–2604, 10.1111/J.1600-6143.2007.01969.X. 17868062

[20] P. J. B. Dyck , A. González‐Duarte , L. Obici , et al., “Development of Measures of Polyneuropathy Impairment in hATTR Amyloidosis: From NIS to mNIS + 7,” Journal of the Neurological Sciences 405 (2019): 1–8, 10.1016/J.JNS.2019.116424. 31445300

[21] V. Di Stefano , P. Guaraldi , A. Romano , et al., “Patisiran in ATTRv Amyloidosis With Polyneuropathy: PatisiranItaly Multicenter Observational Study,” Journal of Neurology 272, no. 3 (2025): 209, 10.1007/S00415-025-12950-3. 39954098 PMC11829936

[22] J. L. De Bleecker , K. G. Claeys , S. Delstanche , et al., “A Retrospective Survey of Patients With Hereditary Transthyretin‐Mediated (hATTR) Amyloidosis Treated With Patisiran in Real‐World Clinical Practice in Belgium,” Acta Neurologica Belgica 123, no. 3 (2023): 1029–1037, 10.1007/S13760-023-02188-Z. 36829087 PMC10238330

[23] L. Gentile , A. Mazzeo , C. Briani , et al., “Long‐Term Treatment of Hereditary Transthyretin Amyloidosis With Patisiran: Multicentre, Real‐World Experience in Italy,” Neurological Sciences 45, no. 9 (2024): 4563–4571, 10.1007/S10072-024-07494-9. 38622453 PMC11306272

[24] M. Dias , L. F. Pinto , M. V. Pinto , et al., “Real‐Life Experience With Inotersen at CEPARM, Hospital Universitário Clementino Fraga Filho, Universidade Federal do Rio de Janeiro,” Arquivos de Neuro‐Psiquiatria 82, no. 4 (2024): 1–7, 10.1055/S-0044-1781463. PMC10997406 38579737

[25] R. K. Patel , A. Ioannou , Y. Razvi , et al., “Sex Differences Among Patients With Transthyretin Amyloid Cardiomyopathy ‐ From Diagnosis to Prognosis,” European Journal of Heart Failure 24, no. 12 (2022): 2355–2363, 10.1002/EJHF.2646. 36575133 PMC10087683

[26] D. Adams , I. L. Tournev , M. S. Taylor , et al., “Efficacy and Safety of Vutrisiran for Patients With Hereditary Transthyretin‐Mediated Amyloidosis With Polyneuropathy: A Randomized Clinical Trial,” Amyloid 30, no. 1 (2023): 18–26, 10.1080/13506129.2022.2091985. 35875890

[27] T. Coelho , W. Marques , N. R. Dasgupta , et al., “Eplontersen for Hereditary Transthyretin Amyloidosis With Polyneuropathy,” JAMA 330, no. 15 (2023): 1448–1458, 10.1001/JAMA.2023.18688. 37768671 PMC10540057

