# Supplementary figures and images for: First‐Generation TTR Silencing Therapies in Hereditary Transthyretin Amyloidosis With Polyneuropathy: Real‐World Insights From a German Single‐Referral Center
Source: Eur J Neurol. 2026 Feb 18;33(2):e70529. doi: 10.1111/ene.70529 (PMC12914347; doi:10.1111/ene.70529)

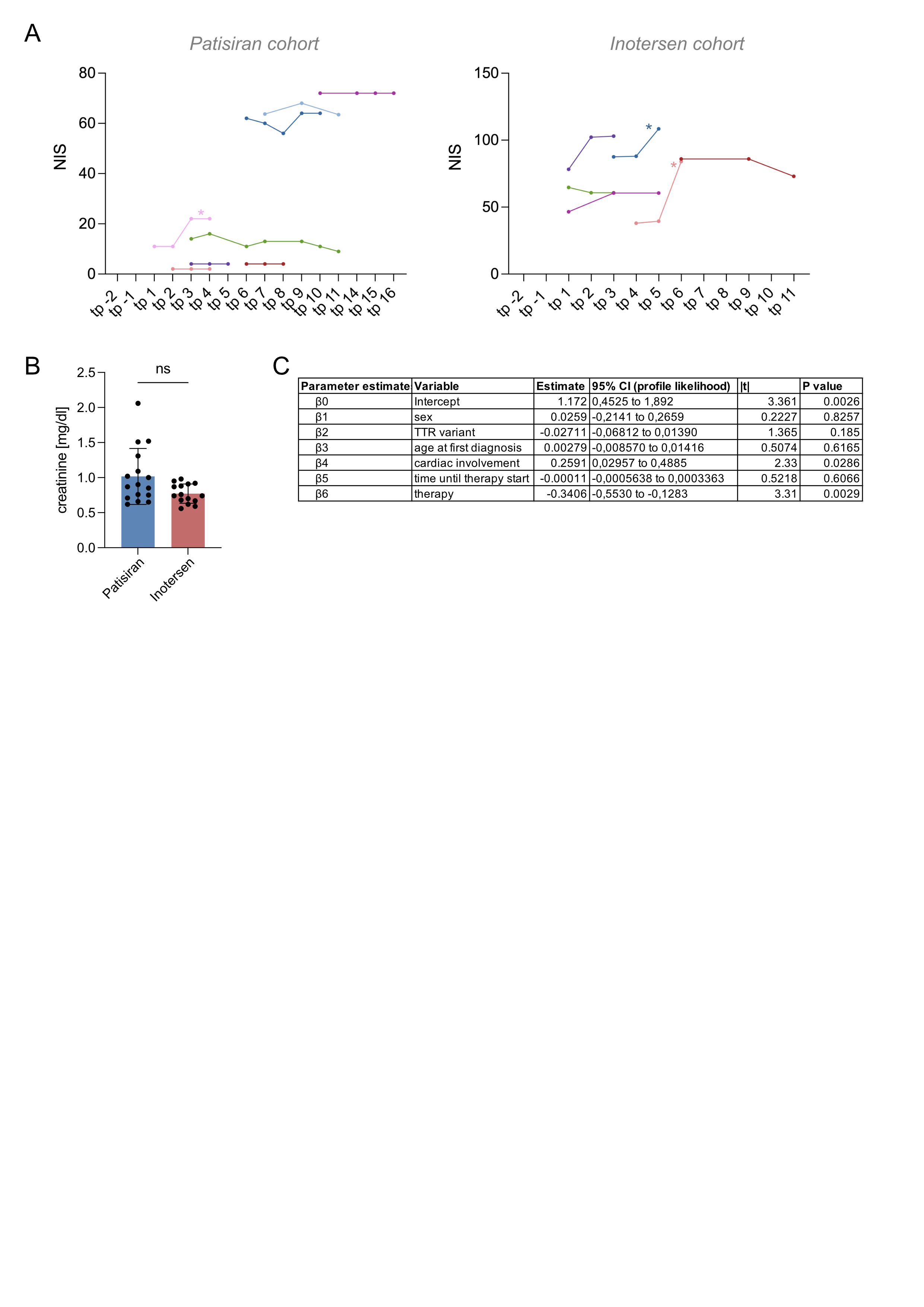

Supplement: Supplementary file 1 — Figure S1: (A) NIS during the course of therapy. Each line presents the temporal development of the NIS per patient. Data were obtained at the following timepoints: tp −2: 2–2.5 years pretreatment, tp −1: 1–1.5 years pre‐treatment, tp 1: baseline, tp 2: 3–7 months, tp 3: 9–15 months, tp 4: 16–21 months, tp 5: 22–27 months, tp 6: 28–31 months, tp 7: 33–38 months, tp 8: 40–42 months, tp 9: 44–48 months, tp 10: 51–53 months, tp 11: 54–57 months, tp 12: 59–60 months, tp 13: 65 months, tp16: 71 months after start of therapy.| (B) Patients treated with patisiran showed higher serum creatinine levels at baseline than patients treated with inotersen, but this difference was not statistically significant (Mann–Whitney‐test, p = 0.0733), patisiran n = 16, inotersen n = 15. (C) Multiple linear regression analysis for serum creatinine levels. Analysis demonstrates that therapy, i.e., patisiran or inotersen, was a significant predictor of creatinine levels (p = 0.0029) after adjustment for sex, genotype, age, cardiac involvement, and time from diagnosis until start of therapy. Additionally, cardiac involvement showed an independent association with serum creatinine levels (p = 0.0286). [file ENE-33-e70529-s001.tiff]
